# Effect of Extrinsic Reward on Motor Plasticity during Skill Learning

**DOI:** 10.1523/ENEURO.0410-24.2025

**Published:** 2025-04-08

**Authors:** Goldy Yadav, Pierre Vassiliadis, Cecile Dubuc, Friedhelm C. Hummel, Gerard Derosiere, Julie Duque

**Affiliations:** ^1^Institute of Neuroscience, Université catholique de Louvain, Brussels 1200, Belgium; ^2^Defitech Chair of Clinical Neuroengineering, Neuro-X Institute (INX), École Polytechnique Fédérale de Lausanne (EPFL), Geneva 1202, Switzerland; ^3^Defitech Chair of Clinical Neuroengineering, Neuro-X Institute (INX), EPFL Valais, Clinique Romande de Réadaptation, Sion 1951, Switzerland; ^4^Clinical Neuroscience, University of Geneva Medical School, Geneva 1202, Switzerland; ^5^Université Claude Bernard Lyon 1, CNRS, INSERM, Centre de Recherche en Neurosciences de Lyon (CRNL), U1028 UMR5292, Impact Team, Bron F-69500, France

**Keywords:** corticospinal excitability (CSE), motor-evoked potentials (MEPs), motor skills, plasticity, primary motor cortex (M1), reinforcement, reward

## Abstract

Human motor skill acquisition is improved by performance feedback, and coupling such feedback with extrinsic reward (such as money) can enhance skill learning. However, the neurophysiology underlying such behavioral effect is unclear. To bridge this gap, we assessed the effects of reward on multiple forms of motor plasticity during skill learning. Sixty-five healthy participants divided into three groups performed a pinch-grip skill task with sensory feedback only, sensory and reinforcement feedback, or both feedback coupled with an extrinsic monetary reward during skill training. To probe motor plasticity, we applied transcranial magnetic stimulation at rest, on the left primary motor cortex before, at an early-training time point, and after training in the three groups and measured motor-evoked potentials from task-relevant muscle of the right arm. This allowed us to evaluate the amplitude and variability of corticospinal output, GABAergic short-intracortical inhibition, and use-dependent plasticity before training and at two additional time points (early and end training). At the behavioral level, monetary reward accelerated skill learning. In parallel, corticospinal output became less variable early on during training in the presence of extrinsic reward. Interestingly, this effect was particularly pronounced for participants who were more sensitive to reward, as evaluated in an independent questionnaire. Other measures of motor excitability remained comparable across groups. These findings highlight that a mechanism underlying the benefit of reward on motor skill learning is the fine-tuning of early-training resting-state corticospinal variability.

## Significance Statement

Skill acquisition is enhanced in the presence of reward. Despite its potential clinical relevance for motor rehabilitation, the underlying neurophysiological mechanisms remain largely unexplored. Specifically, whether reward affects the plasticity of the motor cortex in the context of skill learning is unclear. We show that reward reduces the variability of corticospinal output at an early stage during training and that this effect correlates with individual sensitivity to reward. Our results suggest that a key mechanism underlying the beneficial effect of reward on motor skill learning may be an increase in the stability of motor output in response to training during the early stages of skill learning.

## Introduction

The ability to learn a wide variety of motor skills is a fundamental feature of human behavior, which is associated with plastic reorganization in the motor system (Sampaio-Baptista et al., 2018; Krakauer et al., 2019). Motor skills may emerge from distinct but interindependent learning processes such as explicit strategies (Xeroulis et al., 2007; Wulf et al., 2010), implicit sensorimotor processes (Magill, 1998; Kal et al., 2018), use-dependent plasticity (UDP; Mawase et al., 2017), and reinforcement learning (Lohse et al., 2019; Vassiliadis et al., 2024). Among these, reinforcement learning, which is a well-conserved evolutionary mechanism enabling appropriate action selection based on previous outcomes (Cisek, 2019), appears to play a crucial role in guiding human motor behavior (Dhawale et al., 2017; Vassiliadis and Derosiere, 2020; Vassiliadis et al., 2021). Research indicates that providing reinforcement feedback during training can significantly improve motor skills (Abe et al., 2011; Dayan et al., 2014; Mawase et al., 2017), a finding that holds promise for clinical translation in motor rehabilitation (Widmer et al., 2022).

Reinforcement motor learning has been typically investigated by providing reinforcement feedback (i.e., knowledge of performance such as whether the executed movement was a success or failure) coupled with an extrinsic reward (i.e., value associated with the possible outcomes such as monetary incentives or social praise; Wachter et al., 2009; Abe et al., 2011; Steel et al., 2016; Sporn et al., 2022). An important difference between these two aspects is that reinforcement feedback provides useful information on previous movements to guide future motor adjustments (e.g., moving to a different location after failure), but coupling such feedback with an extrinsic reward does not provide any additional information that may drive learning (e.g., whether a successful reach is rewarded 1 cent or 100 euros does not give any additional information on how to correct the movement per se; Vassiliadis et al., 2021). Providing knowledge of performance improves learning by boosting intrinsic motivation to perform well (Weeks and Kordus, 1998; Thorpe and Valvano, 2002; Oppici et al., 2024) and by allowing the regulation of motor variability in response to movement outcomes (e.g., success or failure; Wu et al., 2014; Therrien et al., 2016; Vassiliadis et al., 2019, 2021). On the other hand, the prospect of reward can provide extrinsic motivation, improve the speed accuracy trade-off of movements, and regulate aspects of motor control, such as feedback control gains (Carroll et al., 2019; De-Comite et al., 2022; Codol et al., 2023), limb stiffness (Codol et al., 2020), or movement fusion (Sporn et al., 2022). Consistent with this specific role of extrinsic reward on motor learning, recent research showed that the combination of reinforcement feedback and monetary incentives improved motor learning compared with when only reinforcement feedback was provided (Vassiliadis et al., 2021; Sporn et al., 2022). However, despite these promising findings at the behavioral level, the effect of extrinsic reward at the neural level remains largely unexplored. It is still unclear how extrinsic rewards during training modulate plasticity in the motor system.

In the present study, we investigated plasticity measures related to excitability changes in the primary motor cortex (M1) during motor skill learning. M1 is a crucial hub of the motor learning network (Reis et al., 2009; Hardwick et al., 2013; Kawai et al., 2015), which undergoes plastic reorganization in the early stages of motor learning (Pascual-Leone et al., 1995; Rioult-Pedotti et al., 1998, 2000, Classen et al., 1998; Butefisch et al., 2004; Duque et al., 2008). Moreover, recent research shows that M1 responds to reward (Ramakrishnan et al., 2017; Levy et al., 2020; Lee et al., 2022) and receives dopaminergic projections from the midbrain that convey reinforcement information during motor learning (Hosp et al., 2011; Leemburg et al., 2018). In addition, learning to adapt movements with knowledge of performance induces plastic changes in M1 (Uehara et al., 2018), including UDP (Mawase et al., 2017), and is boosted when combined with stimulation of M1 (Spampinato et al., 2019). Finally, the presence of reward can increase corticospinal excitability (CSE) in humans (Klein et al., 2012) and modulate GABAergic short-intracortical inhibition (SICI) in M1 (Thabit et al., 2011; Spampinato et al., 2019; Hamel et al., 2023).

Based on these findings linking M1 and reward, we set out to explore skill training-related plasticity in M1 in a subset of individuals (*n* = 65) who participated in a previous study (Vassiliadis et al., 2021) and learned a motor skill task in the presence or absence of reward. We measured the amplitude and variability of CSE, SICI, and UDP at different resting time points during the experiment with a specific focus at an early stage of training, i.e., when groups are exposed to different feedback, with only one group receiving both reinforcement feedback and monetary reward, and at the end of training, i.e., when all groups receive the same reinforcement feedback on the skill task with no monetary reward.

## Materials and Methods

Sixty-five right-handed healthy subjects successfully completed this study (44 females; 23.85 ± 3.22 years old) which involved motor skill learning and neurophysiological measurements using transcranial magnetic stimulation (TMS) over primary motor cortex (M1). As mentioned earlier in the Introduction, the data of these subjects (*n* = 65) were from a larger pool of subjects (*n* = 90) previously exploited in a separate study where we specifically studied behavioral changes underlying motor skill learning associated with monetary reward (Vassiliadis et al., 2021). In this current paper, we focus on motor plasticity measured in the form of motor excitability changes accompanying this form of reward-related skill learning. Subjects filled out a TMS safety questionnaire to look for any contraindications and gave written informed consent in accordance with the ethics committee of the university (approval number 2018/22MAI/219) and the principles of the Declaration of Helsinki. The handedness of these subjects was assessed via the Edinburgh Handedness Inventory (Oldfield, 1971). In addition, all subjects completed a French adaptation of a short version of the Sensitivity to Punishment and Sensitivity to Reward Questionnaire (SPSRQ; Torrubia et al., 2001; Lardi et al., 2008) at the beginning of the experiment. None of the subjects suffered from any neurological disorder or had any history of psychiatric illness and drug or alcohol abuse, and none reported undergoing any drug treatment that could bias their performance or their underlying neural activity. Individuals had normal or corrected vision. All subjects were financially compensated and naive to the purpose of the study.

### Experimental design

#### Motor skill learning task

##### Task apparatus

Subjects were seated in a quiet and dimly lit room, approximately 60 cm in front of a cathode-ray tube computer screen (100 Hz refresh rate). The latter was used to display the motor skill learning task implemented using Matlab 7.5 (MathWorks) and Psychophysics Toolbox extensions (Brainard and Vision, 1997; Pelli, 1997). Subjects were seated with their forearms positioned in prosupination on a table placed in front of them. With the arms in this position, they were able to pinch a manipulandum (Arsalis) with the thumb and index fingers, as required by the task. Subjects were explicitly asked to keep their eyes open, with the gaze oriented toward the screen (not the manipulandum), throughout the entire experiment.

##### Task design

**Figure 1.**
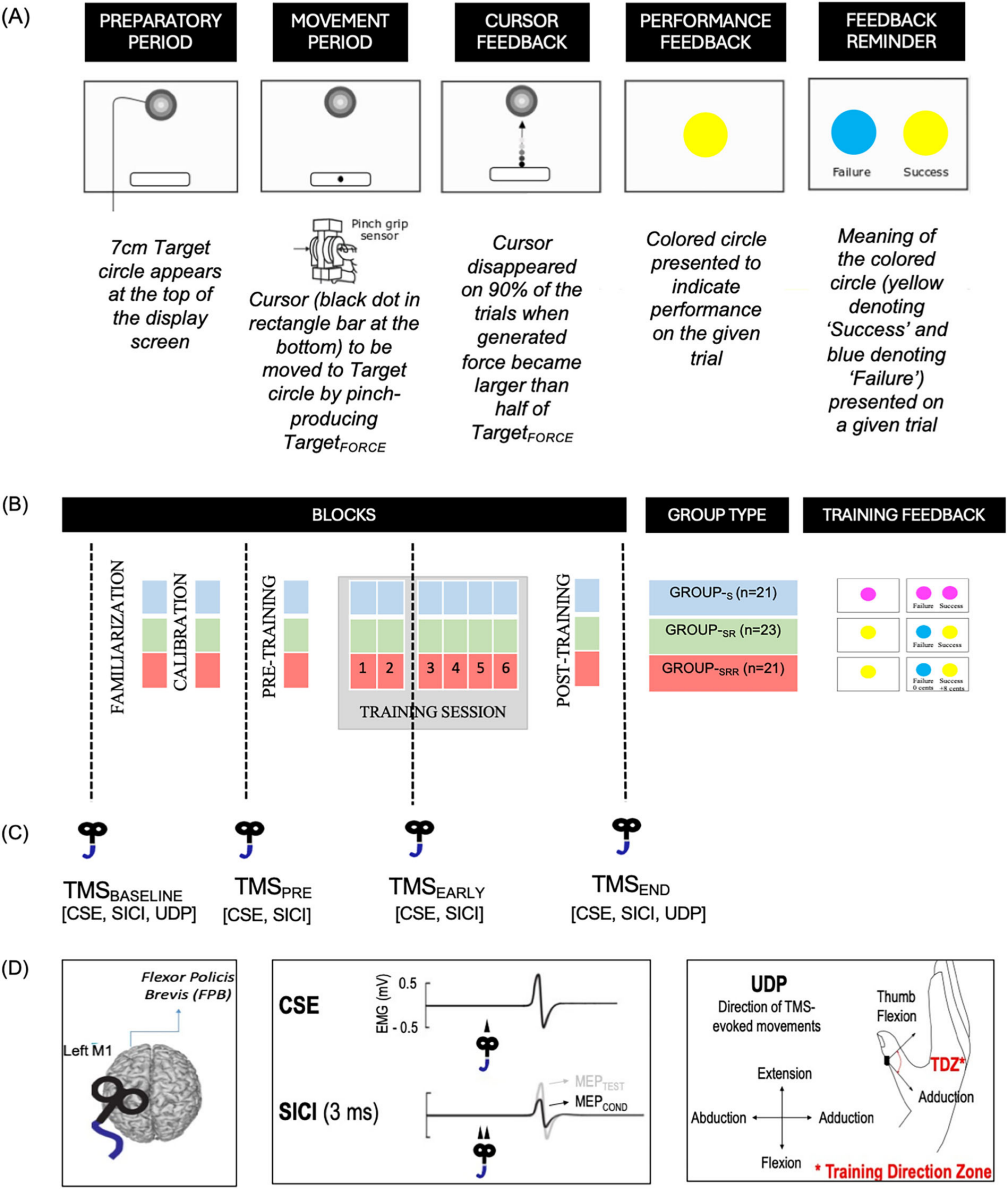
***A***, Motor skill task depicting a target circle (top) and a rectangle start position (bottom). Participants were required to move the cursor (black dot) from the start position to the target circle by pinching the transducer and producing Target_FORCE_. At the end, performance feedback was provided in the form of colored circles (yellow or blue) with a reminder screen presenting the meaning of the colored circle (blue denoting “failure” and yellow denoting “success”). ***B***, Study design depicting all the blocks for the skill task (familiarization, calibration, pretraining, training session, and post-training). All three groups followed the same task design except that during the training session, they received different performance feedback depending on the group type—Group-_S_ (in blue) received only sensory feedback, Group-_SR_ (in green) received sensory and reinforcement feedback, and Group-_SRR_ (in red) received sensory-reinforcement feedback coupled with monetary reward. Note that except for the training session, all three groups received the same performance feedback (sensory reinforcement) on all other blocks. ***C***, TMS measurements (CSE, SICI, UDP) obtained at four different resting time points—TMS_BASELINE_, TMS_PRE_, TMS_EARLY_, and TMS_END_. ***D***, TMS was applied on the left M1 to obtain MEPs from the flexor policis brevis (FPB) muscle to assess CSE (MEP amplitude and variability), SICI (ratio of MEP_CONDITIONED_ and MEP_TEST_), and UDP [TMS-evoked movements elicited in the training direction zone (TDZ), i.e., thumb adduction and flexion in our motor skill task].

The motor skill task consisted of a previously described force modulation paradigm (Vassiliadis et al., 2021, 2022). Briefly, this task required participants to squeeze the force manipulandum using the thumb and index fingers to control a cursor displayed on the screen. Increasing the force resulted in the cursor moving vertically upward (Fig. 1*A*). Each trial started with a “preparatory period” in which a fixed target (7 cm diameter) appeared at the top of the screen and a sidebar (9 × 15 cm) appeared at the bottom. After a variable time interval (0.8–1 s), a black cursor (1 cm diameter) became visible in the sidebar, indicating the start of the “movement period.” Subjects had to pinch the manipulandum in order to move the cursor as quickly as possible from the sidebar to the target and maintain it there for the rest of the movement period lasting for 2 s. The level of force required to reach the target (Target_FORCE_) was individualized for each participant estimated at the start of the experiment and set at 10% of maximum voluntary contraction (MVC). Notably, squeezing the manipulandum before the appearance of the cursor was considered as an anticipation and led to interruption of the trial. Anticipation trials were rare given the variable preparatory period and were discarded from further analyses. On 90% of trials, the cursor disappeared shortly after the start of the movement period when the generated force became larger than half of the Target_FORCE_ (i.e., 5% of MVC). Hence, subjects had to learn to approximate the Target_FORCE_ in the absence of full visual feedback in order to perform appropriately in these partial vision trials. In the remaining 10% of trials, the cursor did not disappear (full vision trials, not included in the analysis). Partial visual feedback was used here in order to increase the impact of other forms of performance feedback (detailed later) on learning (Izawa and Shadmehr, 2011, Mawase et al., 2017, Vassiliadis et al., 2021).

To evaluate task performance, we calculated an “Error” parameter for each trial. The Error was defined as the mean of the difference between the exerted force and the Target_FORCE_ between 0.15 and 2 s after trial onset and was expressed in %MVC. Hence, the more the force differed from the Target_FORCE_, the higher the Error on a given trial. The first 0.15 s were not considered for computing the Error because we assumed that this was the minimum time required for subjects to react to the appearance of the cursor (Steel et al., 2016). A trial was then classified as “success” or “failure” if the Error was under an individualized success threshold (determined during “familiarization” session, detailed next) to provide “performance feedback” in the form of colored circles (along with a “feedback reminder” screen). Importantly, subjects were explicitly told that task success depended on their ability to approximate the Target_FORCE_ as fast and accurately as possible. In summary, for successful task performance (i.e., to have a low Error on this skill task), subjects had to quickly initiate the force and be as accurate as possible in reproducing the Target_FORCE_ to move the cursor to target and maintain it there for 2 s.

##### Task blocks and groups

Subjects first performed a “familiarization” block which consisted of 20 full vision trials (not used for analysis) to allow the subjects to become acquainted with the task. After this block, the remaining blocks consisted of 90% partial vision trials that were considered for analysis. Once familiarized, subjects performed a “calibration” block of 20 trials, which served to individualize the difficulty of the task for each subject for the rest of the experiment. As such, for each individual subject, partial vision trials of the “calibration” block were sorted in terms of Error from the lowest to the greatest in the percentage of MVC. We took the 35th percentile of the Error to determine the individual success threshold (Vassiliadis et al., 2021 for details on the calibration procedure). For the rest of the experiment, trials with an Error lower than this threshold were considered as “success” while trials with higher Error values than this threshold were considered as “failure.” Following “calibration,” subjects performed a total of 280 trials (eight blocks) of the skill task, out of which 20 trials in the beginning and 20 trials at the end served to assess performance on “pretraining” and “post-training” blocks, respectively. At the end of each trial of these blocks (i.e., “familiarization, calibration, pretraining, and post-training”; Fig. 1*B*), subjects were presented with knowledge of performance feedback—a yellow or blue colored circle (presented for 1 s) immediately followed by a reminder screen (presented for 1.5 s) with both colored circles indicating “success” and “failure,” respectively, on a given trial (Fig. 1*A*). In between the “pretraining” and “post-training” blocks, there were six training blocks (Blocks 1–6) of 40 trials each. During this ix-block training session, subjects were divided into three groups, namely, Group_-S_ (*n* = 21), Group_-SR_ (*n* = 23) and Group_-SRR_ (*n* = 21), depending on the performance feedback they received (Fig. 1*B*). Individuals in Group_-S_ received only somatosensory feedback to perform the task during this session: participants were explicitly aware that performance feedback was noninformative (magenta circles were displayed regardless of the performance). Group_-SR_ continued receiving the knowledge of performance feedback in the form of yellow or blue circles (indicating “success” or “failure”). For Group_-SRR_, this knowledge of performance was coupled with a monetary reward—8 cents and 0 cent for “success” and “failure,” respectively, for each trial. Therefore, contrary to Group_-S_, Group_-SR_ and Group_-SRR_ continued receiving knowledge of performance feedback, with this feedback coupled with a monetary reward only in Group_-SRR_ during the six blocks of training. However, note again that irrespective of the group type, all subjects received both sensory and reinforcement feedback for their performance on each trial during the “familiarization, calibration, pretraining, and post-training” blocks. The feedback changed only during the trials of the training session depending on the group type (sensory feedback only for Group_-S_, sensory and reinforcement for Group_-SR_, and sensory, reinforcement, and reward for Group_-SRR_; Fig. 1*B*, training feedback panel). Overall, participants performed a total of 320 trials of the motor skill task (20 trials each during “familiarization, calibration, pretraining, and post-training” blocks, respectively, and 40 trials each on the six blocks of the training session) in this study.

The six training blocks were separated by breaks of 1.5 min to prevent fatigue and by a 7 min TMS session to probe motor plasticity at the end of training block 2 (detailed in the next section). The entire experiment, including the TMS setup, the motor skill task, resting motor plasticity measurements, and the breaks took ∼2.5–3 h for each subject. Finally, subjects received a fixed show-up fee corresponding to 10 euros/h of experiment. In addition, participants also gained a monetary bonus—set at 10 euros for subjects in Group_-SR_ and Group_-S_, while it was variable from 0 to 20 euros according to performance for the Group_-SRR_ (gain of 8 cents per successful trial in training blocks 1–6). Importantly, this bonus for Group_-SRR_ was determined to match that obtained by the other two groups, and finally it corresponded to 9.20 ± 3.29 euros. A *t* test revealed that the total remuneration at the end was not different across the three groups (*t*_(21)_ = −1.11; *p* = 0.28).

#### Transcranial magnetic stimulation (TMS)

##### TMS procedure

As mentioned in the Introduction, our main objective was to assess M1-related neuroplasticity changes underlying reward-based skill learning. To do so, TMS pulses were delivered at four different resting time points during the experiment (Fig. 1*C*), eliciting motor-evoked potentials (MEPs) (1) before task “familiarization” (TMS_BASELINE_), (2) immediately before “pretraining” (TMS_PRE_), (3) early during training session at the end of block 2 (TMS_EARLY_), and (4) after “post-training” block (TMS_END_). Subjects were asked to remain relaxed and motionless with their feet flat on the ground and their eyes open during TMS assessments. TMS was delivered over left M1 through a 70 mm figure-of-eight coil connected to a magnetic stimulator BiStim^2^ (i.e., two Magnetic Stimulator 200^2^ combined through a connecting module, allowing also the delivery of paired-pulses through one coil; Magstim). The coil was placed tangentially on the scalp with the handle oriented toward the back of the head and laterally at a 45° angle away from the midline, approximately perpendicular to the central sulcus. After fitting the participant with a head cap (Electro-Cap, Electro-Cap International), the left M1 “hot spot” was identified by searching the optimal scalp position at which a single pulse of TMS consistently produced detectable motor-evoked potential (MEP) in the contralateral (right) flexor pollicis brevis (FPB), an agonist muscle required in our motor skill learning task. We marked this location on the cap to provide a reference mark throughout the experiment (Vassiliadis et al., 2020; Derosiere et al., 2022; Neige et al., 2023; Wilhelm et al., 2024) and proceeded to determine the resting motor threshold (rMT) for each subject, which corresponds to the minimal TMS intensity required to evoke MEPs of ∼50 µV peak-to-peak in the target muscle (i.e., right FPB) in at least 5 out of 10 consecutive stimulations (Grandjean et al., 2018; Vassiliadis et al., 2018). Furthermore, TMS intensity required to achieve MEPs of 1 mV in FPB was evaluated. Finally, a three-dimensional accelerometer (Kistler Instrument) was fixed on the distal interphalangeal joint of the thumb to determine the direction and amplitude of each TMS-evoked movement to assess use-dependent plasticity at the end of training (UDP, detailed in the next section).

To assess skill learning-related motor plasticity, we applied single-pulse TMS on the M1 hotspot of the FPB muscle at an intensity of 130% of the rMT. This intensity was chosen because it allows to obtain reliable MEPs from the target muscle (Z’Graggen et al., 2009), often in the central part of the recruitment curve (Grandjean et al., 2018), and has been previously shown to induce consistent movements of the thumb for examination of UDP (Mawase et al., 2017). Single-pulse stimulations were delivered to obtain MEPs at the four time points—TMS_BASELINE_ (60 pulses), TMS_PRE_ (20 pulses), TMS_EARLY_ (20 pulses), and TMS_END_ (60 pulses). These MEPs were used to assess changes in corticospinal excitability (all time points) as well as UDP (TMS_BASELINE_ and TMS_END_ time points). The time interval between stimulations was random and ranged from 4.5 to 5.5 s to prevent subjects from anticipating the pulses. Next, we wanted to assess the activity of M1 intracortical circuits throughout skill learning in the different groups of subjects. To do so, we used a paired-pulse TMS protocol on M1, a subthreshold conditioning stimulus (at 80% of the rMT), followed by a suprathreshold test stimulus (at an intensity of 1 mV). With this protocol, the response of the test stimulus can be modified by the conditioning stimulus depending on the time interval between the two stimuli—a decrease in the test stimulus amplitude is generally observed between 2 and 6 ms, which is believed to reflect the activation of the GABAergic inhibitory circuits within M1 and the phenomenon, known as short-latency intracortical inhibition (SICI; Derosiere and Duque, 2020). In this experiment, 15 single pulses (at an intensity of 1 mV) and 15 paired-pulses (conditioning pulse at 80%rMT and test pulse at an intensity of 1 mV with an interval of 3 ms) were delivered in a random order to prevent anticipation and we measured the ratio between MEPs obtained from conditioned (test pulse during paired-pulse trials) and unconditioned (test pulse during single-pulse trials) stimuli (MEP_CONDITIONED_/MEP_TEST_). The time interval between the two subsequent stimuli randomly varied between 4.5 and 5.5 s. These measurements were also obtained at four different resting time points, i.e., TMS_BASELINE_, TMS_PRE_, TMS_EARLY_, and TMS_END_. Finally, for these four time points, we obtained the following motor output measures that allowed us to probe M1 plasticity in the context of skill learning—UDP, MEP (mean and variability), and SICI.

##### Electromyography (EMG) recording

EMG was recorded to measure the peak-to-peak amplitude of MEPs using surface electrodes (Ambu BlueSensor NF-50-K/12/EU, Neuroline, Medicotest) placed over the right FPB, with one electrode placed on the body of the muscle and another on the distal interphalangeal joint; the ground electrode was placed on the styloid process of the ulna. The raw EMG signal was amplified (gain of 1 K), bandpass filtered online (10–500 Hz, Neurolog; Digitimer), notch-filtered (50 Hz; Digitimer D360) and digitized at a sampling rate of 2 kHz (CED 1401-3 ADC12 and Signal 6 software, Cambridge Electronic Design) for offline analysis. Data extraction was performed using Signal 6 and Matlab 2018a (MathWorks), respectively.

### Statistical analysis and endpoint measures of interest

For behavioral performance, we assessed motor skill learning in the 65 subjects who received TMS over M1 (for the original behavioral data set of 91 subjects; Vassiliadis et al., 2021) and compared skill learning across the three groups of subjects (group type: Group_-S_, Group_-SR_, Group_-SRR_). Next, related to the goals of this paper, we assessed three resting TMS-related markers of motor plasticity—MEP amplitudes (mean and variability), SICI, and UDP (Fig. 1*D*). To perform group comparisons, learning-related changes in these measures of interest were separately assessed for two main time points critical to our research objectives, i.e., end-training (TMS_END_) and early-training time point during the training session (TMS_EARLY_). As mentioned in the Introduction, TMS_EARLY_ allowed us to examine motor excitability measures when the three groups were learning the skill task with different conditions during the training session, while TMS_END_ allowed us to examine these measures when all groups were provided the same knowledge of performance feedback (but no reward). Assessing data from these two time points thus enabled us to probe the underlying neurophysiological states depending on the task conditions. For analysis, data obtained for TMS_END_ and TMS_EARLY_ were normalized and expressed in percentage (%) of TMS_PRE_ [except in the case of UDP measure for which TMS-evoked movements at TMS_END_ were directly compared with TMS_BASELINE_; see details in Use-dependent plasticity (UDP)] for performing group comparisons. Additionally, with respect to Group_-SRR_ which received reward during the six-block training session, we computed the sensitivity to punishment and reward score obtained from each subject at the beginning of the experiment (based on the SPSRQ) to perform further correlations.

Statistical analyses were performed using JMP software. Analysis of variance (ANOVA) was the primary statistical test for comparing means of various measures for behavior and neurophysiology data set (continuous and normally distributed dependent variables) obtained for each subject randomly allocated to one of the three groups. The significance level was set at 0.05, and a significant effect on the ANOVA was further assessed by performing Tukey's HSD post hoc tests to make all pairwise comparisons. Effect sizes were reported using partial eta-squared (*η*^2^_p_) measure.

#### Motor skill learning

We evaluated skill learning by computing the rate at which Error decreased over training blocks 1 to 6. To compute this value for each individual, we performed a linear fit of the Error data using the equation: SkillError = *k*(BlockNumber) + *C*, where *k* is slope/rate and *C* is the intercept. We obtained the slope (*k*) and intercept (*C*) values for each subject and compared those for the three groups by performing ANOVA. In addition to the rate of skill learning, we also computed and compared the skill Error at the “post-training” block (in % of “pretraining”) for the three groups using a one-way ANOVA as done previously (Vassiliadis et al., 2021).

#### MEP amplitude (mean and variability)

To assess CSE changes during training, we extracted the peak-to-peak MEP amplitude as well as the root mean square (RMS) of EMG activity in the 200 ms preceding TMS pulse. We removed the first MEP of each block, as well as any MEP preceded by a significant muscular activity (based on RMS, threshold set at RMS >0.02 mV). A total of 0.18% of the MEP trials were removed based on this criterion. Next, we removed outlying trials (±2.5 SD of the mean of each block), corresponding to 2.02% of all trials. After removing these outliers, 97.8% of the MEP data set remained with at least 18 MEPs for each TMS time point for each subject. Next, we performed two analyses on this data set: we calculated the mean MEP amplitude (MEP_MEAN_) and the variability of corticospinal output by computing the coefficient of variation (MEP_CV_ calculated as SD/ MEP_MEAN_; based on Klein Flugge et al., 2013; Vassiliadis et al., 2018). As our goal was to better understand excitability-related changes at two main time points with distinct behavioral requirements in our experiment, we performed one-way ANOVA for group comparisons on MEP_MEAN_ and MEP_CV_ measures, separately for TMS_END_ and TMS_EARLY_ time points (both in %TMS_PRE_). Finally, we evaluated the association between training-related changes in corticospinal excitability variability (MEP_CV_, see Results) and sensitivity to reward and punishment scores with specific focus on the reward group (Group_-SRR_) by performing Pearson’s bivariate correlations.

#### Short-intracortical inhibition (SICI)

As described earlier, to evaluate SICI, we alternated between 15 single-pulse (MEP_TEST_) and 15 paired-pulse (MEP_CONDITIONED_) stimuli. We preprocessed the data the same way as for the single-pulse MEPs leading to the removal of 1.06% of MEP_TEST_ and 0.03% of the MEP_CONDITIONED_ data. Next, we computed SICI by obtaining the ratio (MEP_CONDITIONED_/MEP_TEST_). In this way, a smaller value (<1) indicates intracortical inhibition, and a value of 1 or higher indicates no inhibition at all and even facilitation. We had to remove seven subjects who had a SICI ratio >1 at TMS_BASELINE_ (meaning the absence of the SICI effect) and four participants who did not receive this type of stimulation due to technical issues. We performed further analysis on the remaining 54 subjects. Like MEP amplitude data described above, we assessed learning-related changes in SICI ratios at two main time points, TMS_END_ and TMS_EARLY_ (both in %TMS_PRE_), and performed group comparisons with one-way ANOVA. Thus finally, a value of <100% will indicate more intracortical inhibition whereas a value of ≥100% will indicate less inhibition or even facilitation compared with pretraining SICI values.

#### Use-dependent plasticity (UDP)

UDP manifests as directional changes in TMS-evoked movements that become increasingly similar to those required to execute the task during training [i.e., reaching the training direction zone (TDZ); Mawase et al., 2017]. As mentioned above, UDP was evaluated by means of a three-dimensional accelerometer fixed on the thumb, allowing us to extract the direction and amplitude of each TMS-evoked movement and to calculate the first peak acceleration vector in both the horizontal and vertical axis (respectively corresponding to abduction/adduction and flexion/extension), as done in previous studies (Classen et al., 1998; Duque et al., 2008; Galea and Celnik, 2009; Mawase et al., 2017). As our skill task involved pinching a force sensor, TDZ was defined as a combination of adduction and flexion of the thumb (Fig. 1*D*). Notably, while UDP has been mainly evaluated in the context of ballistic movements (Classen et al., 1998, Duque et al., 2008), a recent work suggests that UDP can also be observed following isometric force modulation training (Mawase et al., 2017). To assess training-related plasticity changes, UDP measures were obtained at two time points: at the beginning (TMS_BASELINE_) and end of the experiment (TMS_END_). We analyzed UDP related to training on our skill task in the three groups by comparing the percentage of the TMS-evoked movements that fell into TDZ at TMS_END_ compared with TMS_BASELINE_ by performing a two-way ANOVA (with time point and group as factors). Note that 5 subjects (out of 65) received the 60 pulses at TMS_PRE_, instead of TMS_BASELINE_. Notably, removing these individuals from the analysis did not change the results. All 65 subjects were therefore included.

## Results

### Behavior

#### The rate of motor skill learning was faster when performance was coupled with reward during the training session

**Figure 2.**
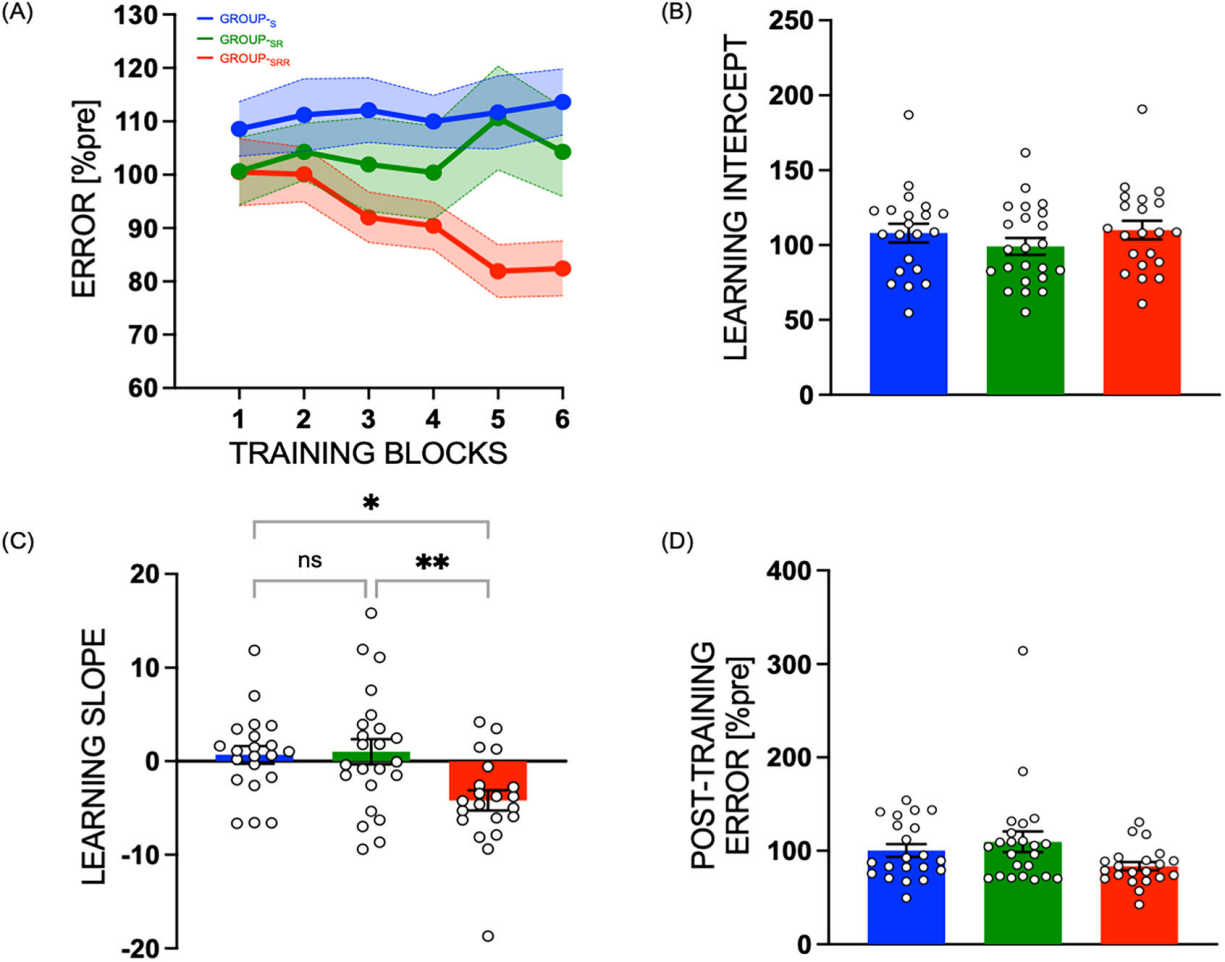
Faster motor skill learning in the presence of reward. ***A***, Motor skill performance of the three groups (Group_-S_, Group_-SR_, Group_-SRR_) during six blocks of training session, with (***B***) no significant difference in learning intercept. ***C***, Rate of learning was significantly different in the group that trained with reward (Group_-SRR_) as compared with the other two groups. ***D***, Post-training error (when all groups perform the task with the same performance feedback) was not significantly different across groups. * indicates *p* < 0.05, ** indicates 0.005 < *p* < 0.05, ns indicates non-significant. Data represent mean, normalized to pretraining block. Error bars/bands represent standard error (SE). White circles (with black outline) represent individual subjects.

We found that skill learning during the training session in the three groups depended on the presence of extrinsic reward during the training session (Fig. 2*A*, Error over Blocks 1–6). To quantify this learning, we computed the intercepts and slopes corresponding to linear fits of these Error data over the six blocks (normalized to pretraining block). For the intercept values (Group_-S_, 108.07 ± 6.16; Group_-SR_, 99.13 ± 5.88; Group_-SRR_. 110.04 ± 6.16; Fig. 2*B*), we found no significant group effect (*F*_(2,62)_ = 0.9428, *p* = 0.3950, *η*^2^_p_ = 0.0295). Hence, all subjects started with a comparable level of performance on the task. Then interestingly, we found a negative slope only for the group that trained with reward (Group_-S_, 0.69 ± 1.17; Group_-SR_, 1.01 ± 1.12; Group_-SRR_, −4.17 ± 1.17] indicating substantial reduction in Error or faster learning rate in Group_-SRR_ (Fig. 2*C*). Statistically, we found a significant effect of group (*F*_(2,62)_ = 6.2759, *p* = 0.0033, *η*^2^_p_ = 0.1683) on the slope. Tukey's post hoc tests revealed that the slope for Group_-SRR_ was significantly higher compared with Group_-SR_ (*p* = 0.0060) as well as Group_-S_ (*p* = 0.0126), while there was no significant difference between Group_-SR_ and Group_-S_ (*p* = 0.9789).

When comparing the Error of groups at the end of the training session on “post-training” block, we found a marginal group effect (*F*_(2,62)_ = 2.6673, *p* = 0.0774, *η*^2^_p_ = 0.07923), with smaller errors for Group_-SRR_ (Group_-S_, 100.39 ± 8.25; Group_-SR_, 109.56 ± 7.89; Group_-SRR_, 83.45 ± 8.25; Fig. 2*D*). Note that this effect was significant in our previous study with a larger sample size (*n* = 91; for more details, see the Results section in Vassiliadis et al., 2021).

### Neurophysiology

#### End-training motor excitability changes

**Figure 3.**
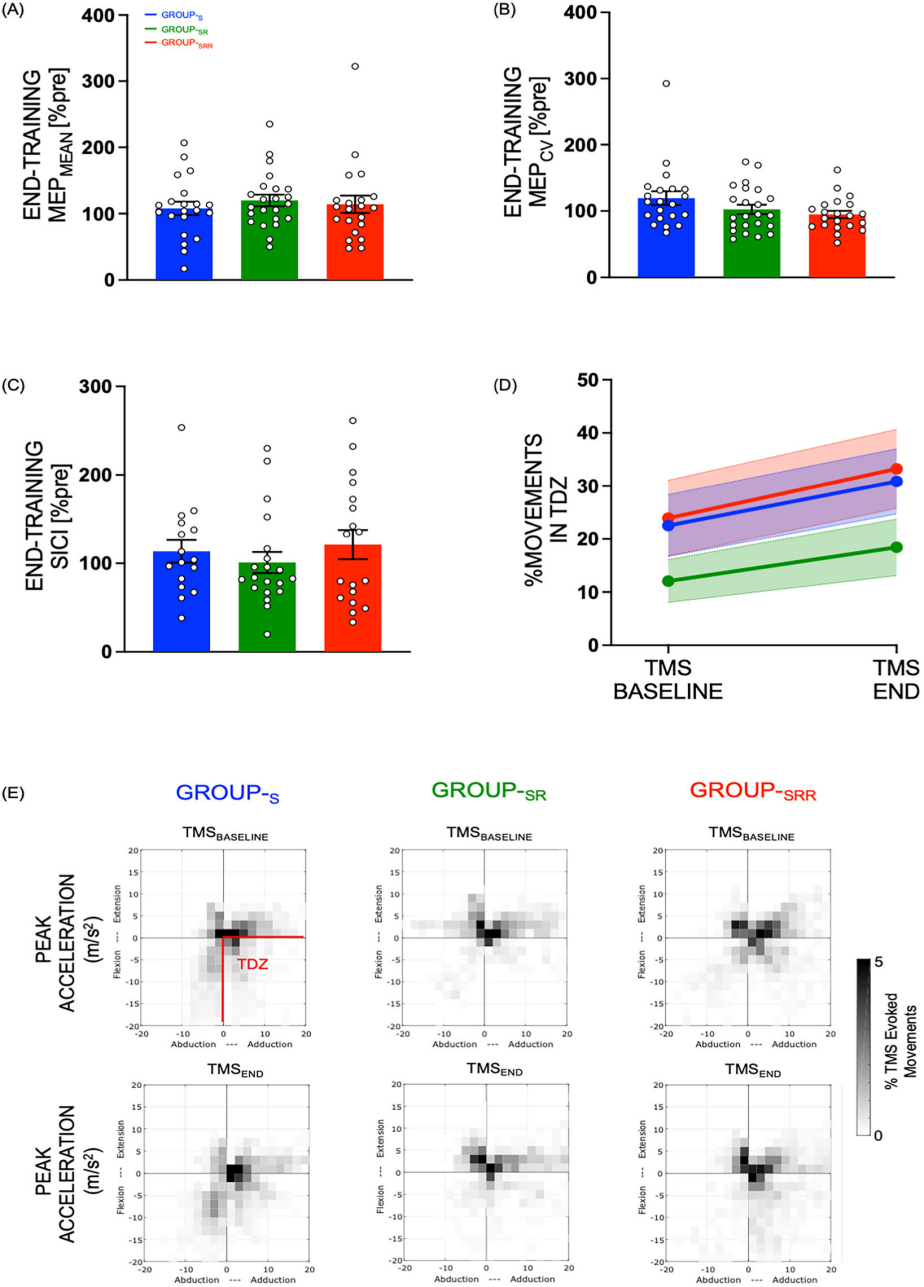
End-training motor plasticity measures—resting TMS measurements obtained after the post-training block when all three groups received the same type of performance feedback on the skill task indicate no significant group differences for (***A***) MEP_MEAN_, (***B***) MEP_CV_, (***C***) SICI, or (***D***) UDP. ***E***, Training-led changes in UDP with more TMS-evoked movements (measured using accelerometers in m/s^2^) obtained at TMS_END_ as compared with TMS_BASELINE_ in all groups in training direction zone (TDZ). Please refer to the gray scale on the right end for interpreting the number of movements in TDZ depicted in the grid plots. Bar plot data represent the mean, normalized to TMS_PRE_. Error bars/bands represent SE. White circles (with black outline) represent individual subjects.

At the end of the training, we obtained resting TMS measurements of MEP (mean amplitude and variability), SICI, and UDP. First, we assessed changes in MEP amplitudes at the end of the training, i.e., TMS_END_ (measured as MEP_MEAN_ and MEP_CV_ and expressed in percentage of TMS_PRE_) in the three groups of subjects. We noted that, at the end of training, all groups displayed MEP_MEAN_ amplitude values above 100% of pretraining (Fig. 3*A*)—Group_-SRR_, 114.15 ± 10.83; Group_-SR_, 119.96 ± 10.35; Group_-S_, 107.91 ± 10.83. However, one-way ANOVA did not reveal any significant effect of group on MEP_MEAN_ (*F*_(2,62)_ = 0.3232, *p* = 0.7251, *η*^2^_p_ = 0.0103). Independent of groups, we noted a significant effect of training on the MEP amplitude (single sample *t* test against 100%: *t*_(64)_ = 2.3294, *p* = 0.0230), suggesting a training-induced increase in MEPs.

On the other hand, for MEP_CV_ at TMS_END_ time point, we noted the lowest variability of MEP in the group that received reward during training session (Group_-SRR_, 94.88 ± 8.02; Group_-SR_, 102.48 ± 7.66; Group_-S_, 119.44 ± 8.02; Fig. 3*B*), with a nonsignificant trend (*F*_(2,62)_ = 2.4618, *p* = 0.0936, *η*^2^_p_ = 0.0735) revealed in the one-way ANOVA. Next, we measured SICI-related changes in the three groups by computing the ratio of MEP_CONDITIONED_ and MEP_TEST_ pulses at the end of training, i.e., TMS_END_ (expressed in percentage of TMS_PRE_). Statistically, we found no significant group effect (*F*_(2,51)_ = 0.5690, *p* = 0.5697, *η*^2^_p_ = 0.0218; Fig. 3*C*; Group_-SRR_, 121.13 ± 13.88; Group_-SR_, 100.97 ± 13.17; Group_-S_, 113.51 ± 14.73). We also pooled the groups together and performed a single sample *t* test against 100% and did not find a significant change in SICI at the end of training (*t*_(53)_ = 1.4349, *p* = 0.1572).

Finally, we assessed UDP and found that the percentage of movements in TDZ was higher at TMS_END_ time point (27.50 ± 3.47) as compared with TMS_BASELINE_ (19.52 ± 3.47; Fig. 3*D*). At the group level, we noted that this value was numerically higher for Group_-SRR_ (28.57 ± 5.54), as compared with Group_-SR_ (15.25 ± 5.29) and Group_-S_ (26.70 ± 5.54). Yet, upon statistical examination, our two-way ANOVA revealed a significant effect of time point (*F*_(1,62)_ = 7.4016, *p* = 0.0084, *η*^2^_p_ = 0.107), but no significant group (*F*_(2,62)_ = 1.7959, *p* = 0.1745, *η*^2^_p_ = 0.055) or time point–group interactions (*F*_(2,62)_ = 0.0885, *p* = 0.9154, *η*^2^_p_ = 0.003). This indicates that skill training led to UDP irrespective of the group type (see Fig. 3*E* for group-wise data of TMS-evoked movements in TDZ).

In conclusion, at the end of training when all groups were back to performing the skill task with the same task performance feedback (no monetary reward and only reinforcement feedback), we did not find any reward-related effects on MEPs, SICI, or UDP for this time point.

#### Early-training motor excitability changes

**Figure 4.**
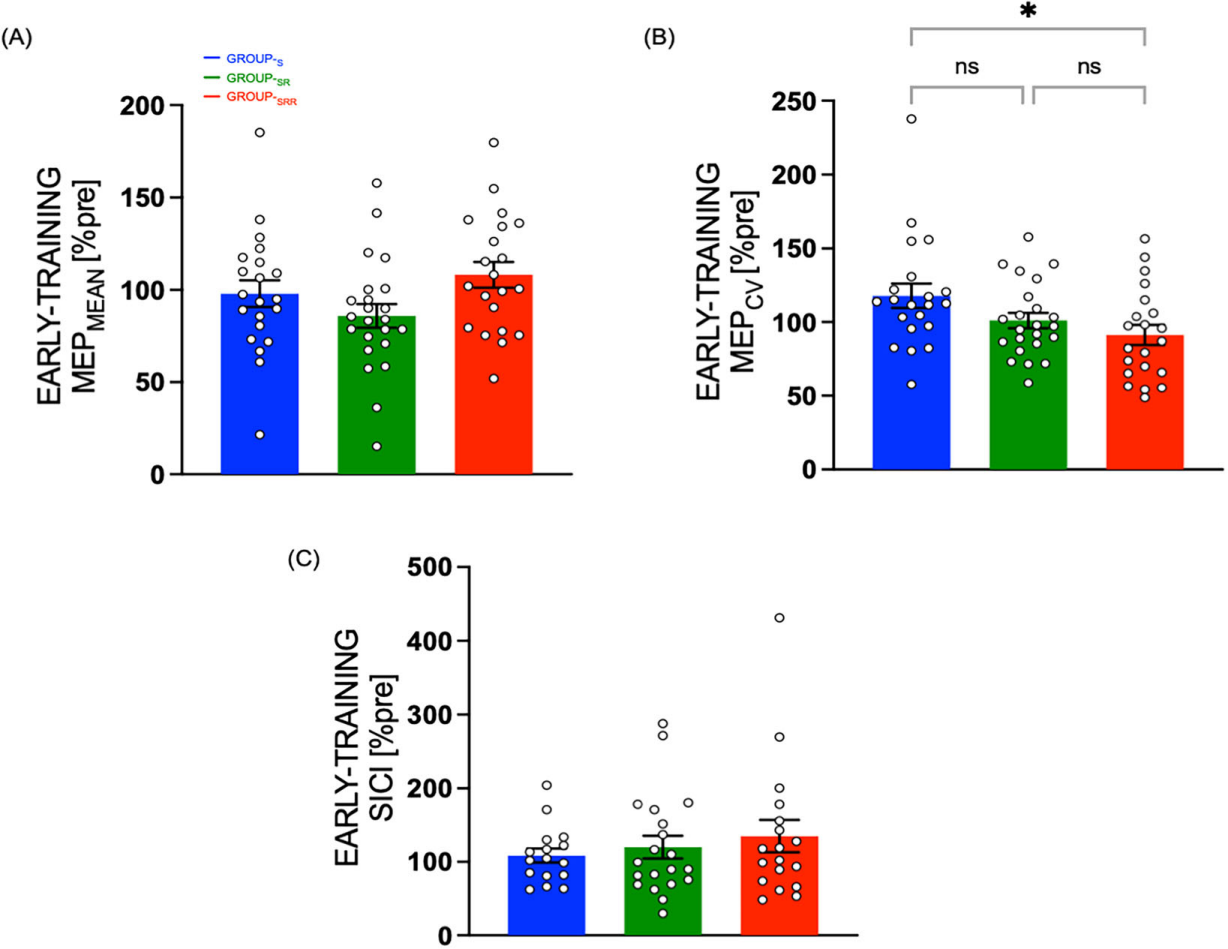
Early-training motor plasticity measures—resting TMS measurements obtained early during training session when the three groups received different types of performance feedback on the skill task indicate (***A***) no significant group differences for MEP_MEAN_. ***B***, MEP_CV_ was significantly lower for Group_-SRR_. ***C***, No group differences observed in SICI. Bar plot data represent the mean, normalized to TMS_PRE_. Error bars represent SE. White circles (with black outline) represent individual subjects.

As mentioned earlier, we were curious to know if exposure to reward influenced motor excitability measures (resting TMS measurements obtained for MEP mean and variability, and SICI) at this early-training time point which differs from the end of training when all groups performed the skill task with the same performance feedback. We, therefore, assessed MEP_MEAN_ and MEP_CV_ values at TMS_EARLY_ (expressed in percentage of TMS_PRE_), which fell at an earlier time point during the training session (between training blocks 2 and 3). Interestingly, at this early time point, there was a marginal group effect for MEP_MEAN_ amplitude (*F*_(2,62)_ = 2.6902, *p* = 0.0758, *η*^2^_p_ = 0.0798) with a trend for larger amplitudes at this early time point observed in the reward group (Fig. 4*A*; Group_-SRR_, 108.18 ± 6.96; Group_-SR_, 85.90 ± 6.65; Group_-S_, 97.98 ± 6.96). Moreover, for MEP_CV_, we noted the lowest variability in the reward group: Group_-SRR_, 91.27 ± 6.90; Group_-SR_, 101.13 ± 6.59; Group_-S_, 117.95 ± 6.90 (Fig. 4*B*). Here, we found a significant effect of group (*F*_(2,62)_ = 3.8217, *p* = 0.0272, *η*^2^_p_ = 0.1097), and follow-up post hoc test revealed a significant difference between Group_-SRR_ and Group_-S_ (*p* = 0.0220), but not between Group_-SRR_ and Group_-SR_ (*p* = 0.5593) or between Group_-SR_ and Group_-S_ (*p* = 0.1913). This effect of reward on MEP variability (when coupled with reinforcement and sensory feedback) particularly pronounced early during training could be consistent with its role in regulating motor variability, as previously shown behaviorally (Dhawale et al., 2017, Vassiliadis et al., 2021). Finally, early-training SICI did not differ between the three groups (Fig. 4*C*; Group_-SRR_, 135.12 ± 16.85; Group_-SR_, 120.28 ± 15.98; Group_-S_, 108.70 ± 17.87; no group effect, *F*_(2,51)_ = 0.5858, *p* = 0.5604, *η*^2^_p_ = 0.0224).

In our protocol, TMS_END_ included more TMS pulses (i.e., 60) than TMS_EARLY_ (minimum 18, see Materials and Methods) to allow us to reliably evaluate UDP when training has ended. Because we found a significant decrease in MEP variability only at TMS_EARLY_, in a control analysis, we asked whether this pattern of results could be explained by the different number of trials at both time points. To do so, we ran the same analyses on bootstrapped MEP values. More specifically, we randomly selected without replacement 18 trials per time point and calculated the mean MEP_MEAN_ and MEP_CV_ over 10,000 resamples. Notably, even when using this approach, we found a similar pattern of results at TMS_END_ for MEP_MEAN_ (*F*_(2,62)_ = 0.3263, *p* = 0.7229, *η*^2^_p_ = 0.0104), with a comparable trend for a group effect in the MEP_CV_ data (*F*_(2,62)_ = 2.5512, *p* = 0.0861, *η*^2^_p_ = 0.0760). This control analysis indicates that the different number of trials per time point cannot explain our findings of lower MEP_CV_ at TMS_END_.

Taken together, the data show an early-training reduction in corticospinal output variability (with no significant effect on mean CSE or GABAergic intracortical inhibition) when participants trained with reward.

#### Correlations between corticospinal excitability measure and sensitivity to reward-punishment scores in the reward group

**Figure 5.**
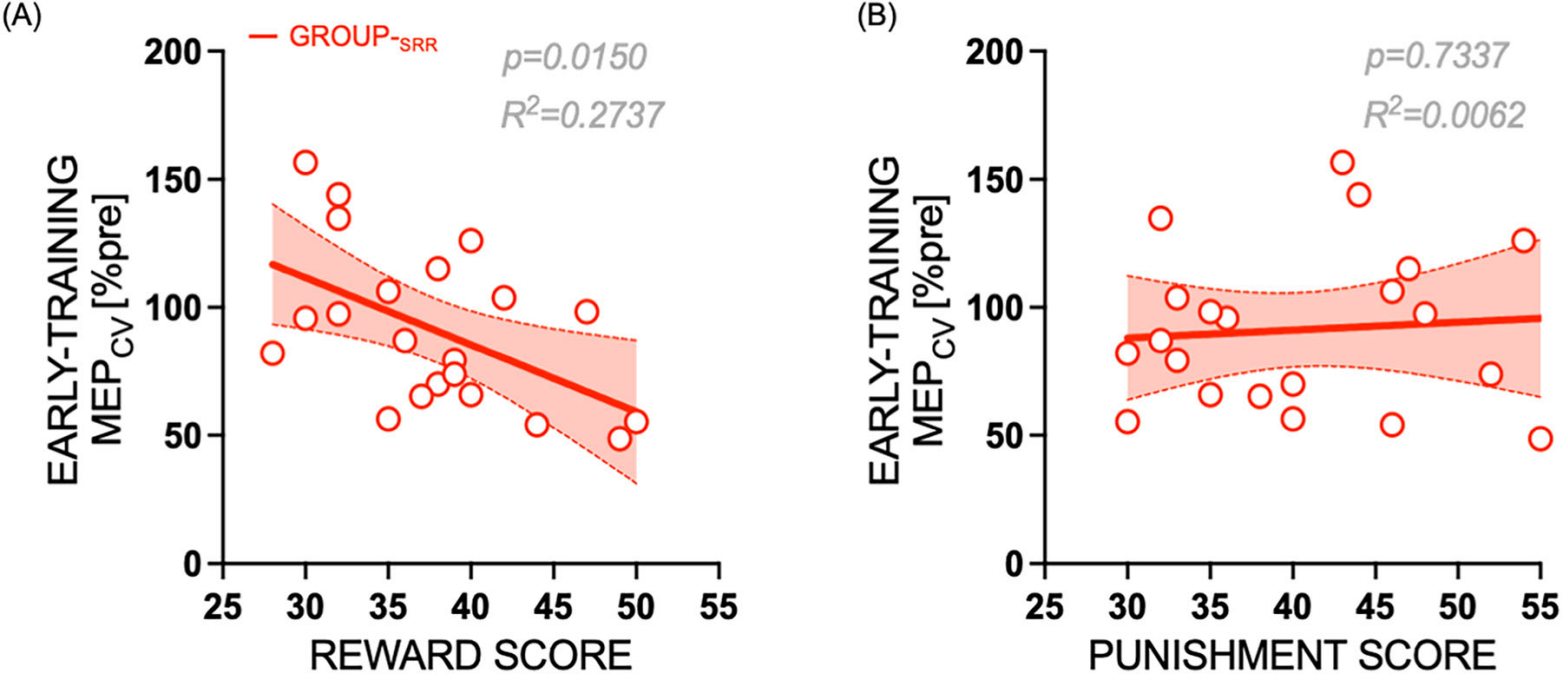
Early-training MEP_CV_ for individuals who received reward during the training session (Group_-SRR_) correlated significantly with (***A***) reward sensitivity scores (individuals with higher reward sensitivity scores have lower MEP variability), but not with (***B***) punishment scores obtained using an independent questionnaire (SPSRQ). Plots show linear regression fit (red solid line) with a 95% confidence interval (red shaded band). White circles (with red outline) represent individual subjects of Group_-SRR_.

In our above assessments on the role of reward on measures of motor plasticity, we found a significant effect of reward on the variability of MEPs, i.e., MEP_CV_ at early-training TMS time point. We therefore wanted to further explore whether this MEP_CV_ obtained at TMS_EARLY_ in the reward group (Group_-SRR_) was associated with the individual sensitivity to reward and punishment measured obtained independently with the SPSRQ (see Materials and Methods). Upon performing bivariate Pearson’s correlation, we found a significant negative correlation between MEP_CV_ and sensitivity to reward scores (*r*(21) = −0.5231, *p* = 0.0150). Note that this effect was not observed for Group_-SR_ (*p* = 0.3361) or Group_-S_ (*p* = 0.1917). On the other hand, for Group_-SRR_, no significant correlation was found between MEP_CV_ and sensitivity to punishment scores (*r*(21) = 0.0789, *p* = 0.7337). This indicates that individuals with higher reward, but not punishment sensitivity scores in Group_-SRR_ had the largest reduction of MEP variability when measured during early training (Fig. 5*A*,*B*). Overall, our findings indicate that reward coupled with performance feedback during training enhances the rate of skill learning with a significant reduction in early-training MEP variability which correlates to individuals’ sensitivity to reward scores.

## Discussion

In this study, we set out to understand the motor neurophysiology underlying the benefits of reward on motor learning. In line with previous work (Vassiliadis et al., 2021; Sporn et al., 2022), we found that the presence of extrinsic reward during training accelerated learning compared with when training was performed with sensory feedback alone or coupled with reinforcement. Next, we assessed the effects of this extrinsic reward on motor plasticity measures by computing CSE (amplitude and variability of MEPs), SICI, and UDP. While we did not find any significant change when evaluating motor plasticity after training, we found that subjects who learned the motor skill in the presence of extrinsic reward exhibited a reduction in CSE variability early on during training. Interestingly, this effect was correlated with individual subjects’ sensitivity to reward scores obtained on self-reported questionnaires. These findings suggest that faster skill learning in the presence of reward is driven by a reduction in the variability of corticospinal output and depends on individual personality traits.

The presence of extrinsic reward significantly reduced CSE variability early during training, and this effect remained at the trend level after learning. This suggests that reward modulates a form of plasticity that is associated with more consistent resting-state CSE, ultimately facilitating skilled motor behavior. As such, neural variability in the motor cortex in monkeys (Churchland et al., 2006a,b) and CSE variability in humans (Klein-Flugge et al., 2013) decreases during action preparation, and this reduction is associated with the efficiency of motor responses. Hence, the reduction of CSE variability we observe might reflect a refinement process that rapidly brings M1 firing rates closer to a specific optimal state for movement generation (Churchland et al., 2006a,b; Klein-Flugge et al., 2013). In line with this idea, computational models suggest that the benefits of reward on motor control rely on a reduction of intrinsic neural noise that could increase the robustness of the corresponding motor representations (Manohar et al., 2015; 2019). Future studies involving electrophysiological recordings during the task are required to better understand the effect of reward on neural noise during motor skill behavior and its relationship to the effects we report at rest.

What neural processes could mediate this reward-driven reduction of neural variability in the motor system? A possible mechanism may be plastic adjustments in circuits connecting hubs of reward processing such as the ventral tegmental area (VTA), the ventral striatum, or the orbitofrontal cortex and M1 (Joel et al., 2002; McHaffie et al., 2006; Berridge, 2012). For instance, there is now evidence showing that M1 reward signals (Ramkumar et al., 2016; Ramakrishnan et al., 2017; Levy et al., 2020), which are modulated during learning (Lee et al., 2022; Ghanayim et al., 2023) and causally involved in reinforcement motor learning (Levy et al., 2020), originate, at least in part, from a dopaminergic pathway linking VTA and M1 (Hosp et al., 2011; Leemburg et al., 2018; Ghanayim et al., 2023). Importantly, VTA–M1 reward signaling seems to be particularly important in the early stages of motor skill acquisition in rodents, but not when a plateau of performance is reached (Hosp et al., 2011, Leemburg et al., 2018), in line with the learning stage-dependent effect of M1 disruption during reward-based decision-making in humans (Derosiere et al., 2017a,b). Consistently, a recent rodent study found that the VTA–M1 pathway was causally involved in the reorganization of M1 in the early stages of a reinforcement motor learning, allowing M1 activity to evolve rapidly toward an expert configuration (Ghanayim et al., 2023). Hence, an interpretation of our result is that the early reduction of corticospinal variability may be related to more consistent neural inputs reaching M1 when training with reward. Interestingly, this effect also scaled with the individual's sensitivity to reward. This is consistent with previous observations that sensitivity to reward (as indexed by the SPSRQ) may reflect interindividual variability in the structure (Barros-Loscertales et al., 2006) and function (Adrian-Ventura et al., 2019) of key hubs of the reward network (e.g., VTA, ventral striatum, and orbitofrontal cortex), possibly modulating their influence on M1 activity during reinforcement skill learning. More research is required to better understand interindividual factors shaping behavioral and neural responsiveness to reward during motor learning, an aspect that could be promising to determine which patients could benefit from reward-based motor rehabilitation protocols. Overall, our data suggest that the early reduction of neural variability in the motor cortex may be an important mechanism underlying the benefits of reward on motor skill learning.

Although we did find a significant increase in CSE amplitude irrespective of the group, in line with previous work (Pascual-Leone et al., 1995; Butefisch et al., 2000; Duque et al., 2008; Galea and Celnik 2009; Christiansen et al., 2018; Vassiliadis et al., 2020), this training-related modulation was not influenced by reward. This result may seem to contrast with previous literature showing reward-related modulations of CSE amplitude in reaction time tasks (Gupta and Aron, 2011; Klein et al., 2012; Freeman et al., 2014; Bundt et al., 2019). Yet, an important difference here is that in these studies, CSE was evaluated during motor preparation within the task, while we measured plasticity at rest, between blocks of training. Hence, a possibility is that reward-related modulations of CSE amplitude during a task do not translate to a persistent change in CSE, in line with a previous study on a smaller sample (Mawase et al., 2017). In this scenario, changes in resting-state CSE amplitude and variability may reflect the operation of distinct mechanisms, possibly reflecting the modulation or either the firing rates of neurons or their consistency during rest (Churchland et al., 2006a). Our results suggest that reward-based motor learning preferentially modulates the resting-state variability of CSE rather than its amplitude. Future work could explore how CSE (variability/amplitude) evolves dynamically during a task, depending on the motivational context and reinforcement feedback received.

In addition to CSE, we investigated the putative effect of reward on SICI and UDP. Indeed, to explore plastic changes reflecting intracortical interactions, we assessed whether GABAergic SICI can be modulated by reward in our task. We observed no reward-driven effect either early during training or at the end of training on SICI. A recent work by Hamel et al. (2023) shows that monetary reward decreases SICI during movement preparation in a motor sequence task, but interestingly such an effect is not observed in their control experiment in which SICI is measured post-movement when the reward feedback has already been processed. This may explain the lack of effect on SICI in our data, where our SICI measurements were made at rest after the training session was over. Our findings imply that intracortical interactions in the presence of extrinsic reward may diminish at rest. Therefore, studies interested in these measurements and reward-based motor skill learning should be designed accordingly. As for UDP, which was measured at the end of training, we noted a practice-induced change (relative to baseline), consistent with prior studies showing plastic changes based on repetition of specific movements toward a target direction (Classen et al., 1998; Duque et al., 2008; Diedrichsen et al., 2010; Huang et al., 2011; Bernardi et al., 2015; Mawase et al., 2017). However, we did not find any group difference related to reward. One plausible explanation is that all three groups in our study repeated the same movement and comparable UDP at the end reflects repetition-based, as opposed to success-based, Hebbian changes in M1 (Bütefisch et al., 2000; Orban de Xivry et al., 2011; Verstynen and Sabes, 2011; see Exp-1 in Mawase et al., 2017). Our results thus do not show an interplay between UDP and extrinsic reward-based reinforcement learning related to M1 for the skill acquired in our study.

Overall, our findings provide neurophysiological support for the incorporation of motivational cues in motor rehabilitation as recently attempted (Therrien et al., 2016, Widmer et al., 2022). More specifically, reward cues could be integrated into innovative technologies for motor restoration such as virtual reality, serious gaming, or rehabilitation robots. In addition, these findings suggest that prescreening patients for reward sensitivity may help to stratify patients according to their responsiveness to a reward-based rehabilitation protocol. Finally, we would like to address some factors that could have influenced our results and the scope of these findings in the context of human motor skill behavior. First, our study could not be fully optimized to isolate subtle changes in UDP. Unlike previous studies on UDP, our force modulation task involved isometric, and not ballistic, movements, and the training direction was not necessarily opposite to the TMS-induced movement direction as classically done in other work (Classen et al., 1998; Duque et al., 2008). Still, our study supports the view that even classical skill force modulation tasks can induce UDP (Mawase et al., 2017). Second, our study involved young healthy participants, and therefore future work is needed to assess if such reward-based effects on neurophysiology can be generalized to other populations such as older adults who typically exhibit reduced learning abilities (Maceira-Elvira et al., 2022). Third, while we utilized monetary reward in our study, the effects of other forms of extrinsic rewards (e.g., social reward; Sugawara et al., 2012) need to be explored further to obtain a better understanding of factors that drive human motor skills. Furthermore, this is not necessarily a limitation but rather a deliberate choice to focus on resting-state measures, and as mentioned earlier, it would be fascinating to explore how CSE evolves dynamically during the task involving a motivational context with reinforcement feedback. Taken together, we report that the presence of reward during skill training induces an early reduction of corticospinal variability at rest during motor skill learning and that this effect depends on an individual's sensitivity to reward. This study supports the view that motivation by reward boosts specific plasticity mechanisms in the motor cortex during motor skill learning and adds to the growing body of literature showing reward-related activity in M1 (Ramakrishnan et al., 2017; Levy et al., 2020). From a broader perspective, these results fit well in the framework of embodied cognition (Foglia and Wilson, 2013; Sullivan, 2018) in which motor behavior and the associated neural mechanisms can be influenced by cognitive processes such as motivation, urgency, emotions, or pain.

## Synthesis

Reviewing Editor: Frederike Beyer, Queen Mary University of London Decisions are customarily a result of the Reviewing Editor and the peer reviewers coming together and discussing their recommendations until a consensus is reached. When revisions are invited, a fact-based synthesis statement explaining their decision and outlining what is needed to prepare a revision will be listed below. The following reviewer(s) agreed to reveal their identity: Armin Torbati. Note: If this manuscript was transferred from JNeurosci and a decision was made to accept the manuscript without peer review, a brief statement to this effect will instead be what is listed below.

Reviewer 1

The study is very interesting, well designed, clearly written and of high relevance in the field of motor control.

While several recent studies have assessed the different effects of feedback type on motor skill learning, the neuroscientific evidence was lagging. Here, the authors use a series of validated TMS protocols to assess skill-learning related plasticity in M1. The findings are specific, showing that reward feedback (+ reinforcement + sensory feedback: SSR group) significantly reduces early-training CSE variability, assessed with the coefficient of variation of MEP (MEP_cv). And this measure is correlated with individual reward sensitivity in the SSR group (N = 21). This reduction in CSE variability suggests a mechanism by which reward stabilises motor output early in training, promoting enhanced skill acquisition, and highlights the differential effects of feedback types on motor cortex plasticity and skill learning.

I have mainly a few minor queries that I would like the authors to address, before the study is published.

Most are very minor, but there is an option to conduct an analysis, if possible and the trial number allows, which would provide exciting evidence regarding dynamic CSE changes during skill learning as a function of success/failure feedback (applicable to SSR and SR groups only during Training).

MINOR:

## Methods: Task.

The trial structure could be slightly clarified.

Figure 1 is useful in showing the blocks of the experimental design.

And the text indicates that e.g. Familiarization block had 20 trials, and Calibration also had 20 trials?

Training seems to have 280 trials, 20 preTraining, 240 Training (6*40 = 240), 20 postTraining.

That means there are 20 + 20 + 20 + 240 + 20, correct? 320?

What was the duration of the experiment?

Including breaks and TMS phases?

This would help researchers design follow-up studies, e.g. considering whether more blocks/trials would be possible or not.

I found this unclear:

"At the end of each trial of these blocks (i.e., Familiarization, Calibration, Pre-Training and Post-Training, see Figure 1B), subjects ..."

Does this mean that participants got feedback only trial 20 of Familiarization, trial 20 of Calibration ,etc?

But feedback (s, sr, srr) on every trial of Training? This could be explained more explicitly. I got there eventually but perhaps a brief explicit mention would help.

## Results

"The intercept values (Figure 2B) were comparable across all three groups

[Group-S: 108.07{plus minus}6.16, Group-SR: 99.13{plus minus}5.88, Group-SRR: 110.04{plus minus}6.16] as indicated by

no significant group effect (F2,62=0.9428, p=0.3950, η2p=0.0295)."

Using frequentist statistics only allows the authors to state that they did not find significant differences, but they have no evidence that the DV is comparable across groups. To make such a statement, the authors would need to conduct Bayesian statistics (proper Bayesian inference or at least Bayes Factor analysis). Please amend the statement regarding "comparable values"

- Typo: "This pattern of result"? Replace with "This pattern of results" (p. 21)

- Please replace "associated to" with "associated with" in the Discussion (p.24, several instances)

MINOR BUT OPTION TO CONDUCT AN ADDITIONAL ANALYSIS, WHICH COULD FEED INTO THE DISCUSSION

- MEP_CV analysis:

Reward and reinforcement feedback were binary in the SR and SRR groups during training.

What is the prediction regarding CSE changes across the SR and SRR groups as a function of success/failure feedback?

Do the authors expect the MEP_cv outcome variable to change in trials following success and failure?

Would it be possible to analyse this? I imagine there may not be enough trials, but it would be interesting to check.

How many success/failure trials are available for MEP_cv at TMS_EARLY (minimum 18 trials) and separately at TMS_END (60 trials)?

Dhawale et al. (2019) analysed 'motor variability' using sets of 5 trials in a running window, so potentially a small number of failure/success trials could help clarify this question. I am aware that signal2noise ratio in trial-wise TMS MEP values will be higher than for the behavioural variables in Dhawale et al (2019), but the outcomes could help clarify whether there is a dynamic modulation of M1 CSE as a function of performance outcomes (feedback about success and failure).

- Learning rates across trials.

Although estimating slope and intercept for each participant separately is acceptable, this approach is less sensitive than hierarchical mixed-model analyses. For instance, modelling DV (ERROR%) as a function of block or trial number, with group as a fixed factor and subject as random effects on slope and intercept, would increase the sensitivity of the analysis to potential group effects on slope and intercepts. The authors might consider these analyses in future work

Reviewer 2

Abstract

Lacks specific details on the neurophysiological measures used, which could provide a clearer understanding of the study's focus on motor plasticity.

Introduction

More explicitly outlines how monetary rewards are hypothesized to affect motor plasticity differently from other types of feedback.

Methods

The inclusion of more specific justifications for the chosen stimuli intensities, and the selection of motor output measures would enhance this section.

The statistical analysis subsection is thorough, but it lacks a discussion on the assumptions of the chosen statistical tests and how these assumptions were tested or met.

Results

Explain why certain changes, for example, in MEP variability are significant and how they relate to motor learning.

Discussion

Explain the clinical benefits of your work. For example, how could these findings be applied in rehabilitation settings? Discuss how rewards could be tailored to individual sensitivity levels to optimize recovery.

Address the limitations more thoroughly

## Author Response

Dear Dr. Beyer, Thank you for providing us the opportunity to revise the manuscript. We are pleased to receive an overall positive assessment of our paper and the constructive feedback provided by the reviewers. We have now addressed the remaining concerns raised by the reviewers. We are hopeful that our response and the corresponding changes in the manuscript make it worthy of publication in eNeuro. We thank you and the reviewers for their time.

Please see below our point-to-point responses to the reviewers' feedback. The reviewers' comments are in bold, our response is in regular text and changes made in the manuscript are highlighted in red.

Synthesis of Reviews:

Reviewer 1 1) The study is very interesting, well designed, clearly written and of high relevance in the field of motor control.

While several recent studies have assessed the different effects of feedback type on motor skill learning, the neuroscientific evidence was lagging. Here, the authors use a series of validated TMS protocols to assess skill-learning related plasticity in M1. The findings are specific, showing that reward feedback (+ reinforcement + sensory feedback: SSR group) significantly reduces early-training CSE variability, assessed with the coefficient of variation of MEP (MEP_cv). And this measure is correlated with individual reward sensitivity in the SSR group (N = 21). This reduction in CSE variability suggests a mechanism by which reward stabilises motor output early in training, promoting enhanced skill acquisition, and highlights the differential effects of feedback types on motor cortex plasticity and skill learning.

Authors' response: Thank you very much for the encouraging comments.

2) I have mainly a few minor queries that I would like the authors to address, before the study is published. Most are very minor, but there is an option to conduct an analysis, if possible and the trial number allows, which would provide exciting evidence regarding dynamic CSE changes during skill learning as a function of success/failure feedback (applicable to SSR and SR groups only during Training).

Authors' response: We acknowledge that this would have been an intriguing analysis; however, our study design does not support such an analysis, assuming we have correctly understood the suggestion. Indeed, all TMS measurements in our study, including CSE changes, were obtained at rest and not while the subjects performed the skill task under different feedback conditions. It is therefore not possible with our current dataset to examine dynamic CSE changes during skill learning as a function of success/failure feedback. We have made efforts to clarify this aspect of the design in several sections of the revised manuscript.

We state:

In the Abstract (also based on point 1 of Reviewer 2): "To probe motor plasticity, we applied transcranial magnetic stimulation at rest, on the left primary motor cortex before, at an early training time-point and after training in the three groups and measured Motor Evoked Potentials from task relevant muscle of the right arm." In the Introduction:

Page 5: "We measured amplitude and variability of CSE, SICI and UDP at different resting time points during the experiment with a specific focus at an early stage of training- i.e., when groups are exposed to different feedback, with only one group receiving both reinforcement feedback and monetary reward - and at the end of training- i.e., when all groups receive the same reinforcement feedback on the skill task with no monetary reward. " In the Methods:

Page 11: "These measurements were also obtained at four different resting time points, i.e., TMSBASELINE, TMSPRE, TMSEARLY and TMSEND." Page 12: "Next, related to the goals of this paper we assessed three resting TMS-related markers of motor plasticity- MEP amplitudes (mean and variability), SICI and UDP." In the Legend of Figure 1C: "TMS measurements (CSE, SICI, UDP) obtained at four different resting time points- TMSBASELINE, TMSPRE, TMSEARLY, TMSEND." And in the Discussion:

Page 26: "Future work could explore how CSE (variability/amplitude) evolves dynamically during a task, depending on the motivational context and reinforcement feedback received." MINOR: ## Methods: Task.

3) The trial structure could be slightly clarified.

Figure 1 is useful in showing the blocks of the experimental design.

And the text indicates that e.g. Familiarization block had 20 trials, and Calibration also had 20 trials? Training seems to have 280 trials, 20 preTraining, 240 Training (6*40 = 240), 20 postTraining. That means there are 20 + 20 + 20 + 240 + 20, correct? 320? What was the duration of the experiment? Including breaks and TMS phases? This would help researchers design follow-up studies, e.g. considering whether more blocks/trials would be possible or not.

Authors' response: There were a total of 320 trials of the skill task. Overall the duration of the experiment was 2.5-3 hours, including TMS set-up, the skill task, TMS measurements and the breaks.

We have clarified this in our manuscript.

We state :

Page 9: "Overall, participants performed a total of 320 trials of the motor skill task (20 trials each during Familiarization, Calibration, Pre-Training and Post-Training blocks respectively, and 40 trials each on the six blocks of the training session) in this study." Page 9: "The entire experiment, including TMS setup, the motor skill task, resting motor plasticity measurements and the breaks, took around 2.5 - 3 hours for each subject." 4) I found this unclear: "At the end of each trial of these blocks (i.e., Familiarization, Calibration, Pre-Training and Post-Training, see Figure 1B), subjects ..." Does this mean that participants got feedback only trial 20 of Familiarization, trial 20 of Calibration ,etc? But feedback (s, sr, srr) on every trial of Training? This could be explained more explicitly. I got there eventually but perhaps a brief explicit mention would help.

Authors' response: Thank you for this comment that allowed us to clarify this aspect. We now provide explicit clarification for this at the end of the paragraph containing the aforementioned statement.

We state on page 9: "However, note again that irrespective of the group type all subjects received both sensory and reinforcement feedback for their performance on each trial during the Familiarization, Calibration, Pre-Training and Post-Training blocks. The feedback changed only during the trials of the training session depending on the group type (sensory feedback only for Group-S, sensory+reinforcement for Group-SR, and sensory+reinforcement+reward for Group-SRR; see Figure 1B-Training Feedback panel)." ## Results 5) "The intercept values (Figure 2B) were comparable across all three groups [Group-S: 108.07{plus minus}6.16, Group-SR: 99.13{plus minus}5.88, Group-SRR: 110.04{plus minus}6.16] as indicated by no significant group effect (F2,62=0.9428, p=0.3950, η2p=0.0295)." Using frequentist statistics only allows the authors to state that they did not find significant differences, but they have no evidence that the DV is comparable across groups. To make such a statement, the authors would need to conduct Bayesian statistics (proper Bayesian inference or at least Bayes Factor analysis). Please amend the statement regarding "comparable values".

Authors' response: We agree with the reviewer and thus we have made the correction in this statement in our manuscript.

We state on page 17: "For the intercept values [Group-S: 108.07{plus minus}6.16, Group-SR: 99.13{plus minus}5.88, Group-SRR: 110.04{plus minus}6.16; see Figure 2B], we found no significant group effect (F2,62=0.9428, p=0.3950, η2p=0.0295)." 6) - Typo: "This pattern of result"? Replace with "This pattern of results" (p. 21) - Please replace "associated to" with "associated with" in the Discussion (p.24, several instances) Authors' response: Thank you for pointing this out. We have made these corrections on page 21 and 24.

MINOR BUT OPTION TO CONDUCT AN ADDITIONAL ANALYSIS, WHICH COULD FEED INTO THE DISCUSSION 7) - MEP_CV analysis:

Reward and reinforcement feedback were binary in the SR and SRR groups during training.

What is the prediction regarding CSE changes across the SR and SRR groups as a function of success/failure feedback? Do the authors expect the MEP_cv outcome variable to change in trials following success and failure? Would it be possible to analyse this? I imagine there may not be enough trials, but it would be interesting to check. How many success/failure trials are available for MEP_cv at TMS_EARLY (minimum 18 trials) and separately at TMS_END (60 trials)? Dhawale et al. (2019) analysed 'motor variability' using sets of 5 trials in a running window, so potentially a small number of failure/success trials could help clarify this question. I am aware that signal2noise ratio in trial-wise TMS MEP values will be higher than for the behavioural variables in Dhawale et al (2019), but the outcomes could help clarify whether there is a dynamic modulation of M1 CSE as a function of performance outcomes (feedback about success and failure).

Authors' response: Thank you again for this interesting point. As mentioned above in point 2), our TMS measurements were obtained at rest and therefore the current dataset cannot address this question. This could be explored in a future study with a task design that allows TMS measurement on a trials by trial basis, thus helping us better understand change in CSE following a success or failure movement feedback-type, as now stated in the Discussion:

Page 26: "Future work could explore how CSE (variability/amplitude) evolves dynamically during the task, depending on the motivational context and reinforcement feedback received" 8) - Learning rates across trials.

Although estimating slope and intercept for each participant separately is acceptable, this approach is less sensitive than hierarchical mixed-model analyses. For instance, modelling DV (ERROR%) as a function of block or trial number, with group as a fixed factor and subject as random effects on slope and intercept, would increase the sensitivity of the analysis to potential group effects on slope and intercepts. The authors might consider these analyses in future work Authors' response: Thank you for this insight that indeed could steer the analyses in future work.

To verify that our results were replicable with the analysis suggested by the reviewer, we ran a linear-mixed effect model on the Error data with the lme package on R. As proposed, we added block and group-type as fixed effects and subject as a random effect on slope and intercept. In line with the initial analysis, we found a significant block x group-type interaction (F2,62 = 7.988, p = 0.0008, η2p = 0.20) with Tuckey-corrected post-hoc tests revealing that the slope for Group-SRR was significantly different compared to Group-SR (p=0.0022) as well as Group-S (p=0.0032), while there was no significant difference between Group-SR and Group-S (p=0.9789). Moreover, intercepts were not different (all p > 0.44). These results show faster learning rate in Group-SRR as compared to the other two groups, as in the main analyses.

These results are reassuring as they show that the reported findings are independent of the statistical approach exploited. With this in mind, we suggest keeping the existing result on slope and intercept to maintain consistency with the rest of the statistical analyses involving repeated-measures ANOVA in our manuscript. We will surely consider the recommended analyses in our future work.

Reviewer 2 Abstract 1) Lacks specific details on the neurophysiological measures used, which could provide a clearer understanding of the study's focus on motor plasticity.

Authors' response: Thank you for pointing this out. We have added some details to this part.

We now state (Abstract, page 2): "To probe motor plasticity, we applied transcranial magnetic stimulation at rest, on the left primary motor cortex before, at an early training time-point and after training in the three groups and measured Motor Evoked Potentials from task relevant muscle of the right arm. This allowed us to evaluate the amplitude and variability of corticospinal output, GABA-ergic short-intracortical inhibition and use-dependent plasticity before training and at two additional time points (early- and end-training)." Introduction 2) More explicitly outlines how monetary rewards are hypothesized to affect motor plasticity differently from other types of feedback.

Authors' response: Thank you for this comment. Our study builds on two lines of prior research - one related to reward influencing reinforcement skill learning (Vassiliadis et al., 2021; Sporn et al., 2022), and the second related to reward influencing M1 activity in various species and tasks (Ramakrishnan et al., 2017; Levy et al., 2020; Lee et al., 2022, Klein et al., 2012). Building on this body of work, this study was primarily exploratory attempting to assess if coupling monetary reward with motor skill performance could have any effect on the underlying resting motor plasticity measures.

While we expected that monetary reward would improve skill learning and modulate the underlying neuroplastic changes, we did not set up a priori hypotheses linking reward and the specific plasticity measures we obtained. The reason for this is that previous TMS literature investigating reward processing in M1 has mostly evaluated activity during specific task periods (e.g., during action preparation see Klein et al., 2012 for CSE amplitude; Hamel et al., 2023 for SICI). Hence, while this type of results do suggest that M1 is sensitive to reward, as largely shown in animals (Ramakrishnan et al., 2017; Levy et al., 2020; Lee et al., 2022, Smoulder et al., 2024), they do not really allow to extrapolate regarding learning-related resting-state changes. We therefore did not provide any explicit hypothesis in the Introduction, but rather described the previous literature linking different measures of M1 activity and reward processing to support the rationale of our study.

We state (Introduction, Page 5): "Based on these findings linking M1 and reward, we set out to explore skill training related plasticity in M1 in a subset of individuals (n=65) who participated in Vassiliadis et al. (2021) and learned a motor skill task in the presence or absence of reward." Methods 3) The inclusion of more specific justifications for the chosen stimuli intensities, and the selection of motor output measures would enhance this section.

Authors' response: We have provided more specific details related to the chosen intensity and motor output measures.

We state (page 11): "To assess skill learning related motor plasticity, we applied single-pulse TMS on the M1 hotspot of the FPB muscle at an intensity of 130% of the rMT. This intensity was chosen because it allows to obtain reliable MEPs from the target muscle (Z'Graggen et al., 2009), often in the central part of the recruitment curve (Grandjean et al., 2018) and has been previously shown to induce consistent movements of the thumb for examination of UDP (Mawase et al., 2017)." We further state (page 11): "Finally, for these four time points we obtained the following motor output measures that allowed us to probe M1 plasticity in the context of skill learning- UDP, MEP (mean and variability) and SICI." 4) The statistical analysis subsection is thorough, but it lacks a discussion on the assumptions of the chosen statistical tests and how these assumptions were tested or met.

Authors' response: Thank you for this point. We have now added information on assumptions of the chosen statistical test in our manuscript.

We state (page 13): "Analysis of variance (ANOVA) was the primary statistical test for comparing means of various measures for behavior and neurophysiology dataset (continuous and normally distributed dependent variables) obtained for each subject randomly allocated to one of the three groups. Significance level was set at 0.05, and a significant effect on the ANOVA was further assessed by performing Tukey's HSD post-hoc tests to make all pairwise comparisons. Effect sizes were reported using partial eta-squared (η2p) measure." Results 5) Explain why certain changes, for example, in MEP variability are significant and how they relate to motor learning.

Authors' response: We have reflected on this in depth and have refined the following statements in our Discussion section in which we now state:

Page 24: "The presence of extrinsic reward significantly reduced CSE variability early during training, and this effect remained at the trend level after learning. This suggests that reward modulates a form of plasticity that is associated with more consistent resting-state CSE, ultimately facilitating skilled motor behavior. As such, neural variability in motor cortex in monkeys (Churchland et al. 2006a, 2006b) and CSE variability in humans (Klein-Flugge et al., 2013) decreases during action preparation and this reduction is associated with the efficiency of motor responses. Hence, the reduction of CSE variability we observe might reflect a refinement process that rapidly brings M1 firing rates closer to a specific optimal state for movement generation (Churchland et al. 2006a, 2006b; Klein-Flugge et al., 2013). In line with this idea, computational models suggest that the benefits of reward on motor control rely on a reduction of intrinsic neural noise that could increase the robustness of the corresponding motor representations (Manohar et al., 2015; 2019). Future studies involving electrophysiological recordings during the task are required to better understand the effect of reward on neural noise during motor skill behavior and its relationship to the effects we report at rest." We further discuss the neural basis of such reward-dependent modulation of CSE on page 24-25: "What neural processes could mediate this reward-driven reduction of neural variability in the motor system? A possible mechanism may be plastic adjustments in circuits connecting hubs of reward processing such as the ventral tegmental area (VTA), the ventral striatum or the orbitofrontal cortex and M1 (Joel et al., 2002; McHaffie et al., 2006; Berridge, 2012). For instance, there is now evidence showing that M1 reward signals (Ramkumar 2016; Ramakrishnan et al., 2017; Levy et al., 2020) which are modulated during learning (Lee et al., 2022; Ghanayim et al., 2023) and causally involved in reinforcement motor learning (Levy et al., 2020), originate, at least in part, from a dopaminergic pathway linking VTA and M1 (Hosp et al., 2011; Leemburg et al., 2018; Ghanayim et al., 2023). Importantly, VTA-M1 reward signalling seems to be particularly important in the early stages of motor skill acquisition in rodents, but not when a plateau of performance is reached (Hosp et al., 2011, Leemburg et al., 2018); in line with the learning stage-dependent effect of M1 disruption during reward-based decision-making in humans (Derosiere et al., 2017a, 2017b). Consistently, a recent rodent study found that the VTA-M1 pathway was causally involved in the reorganization of M1 in the early stages of a reinforcement motor learning, allowing M1 activity to evolve rapidly towards an expert configuration (Ghanayim et al., 2023). Hence, an interpretation of our result is that the early reduction of corticospinal variability may be related to more consistent neural inputs reaching M1 when training with reward. Interestingly, this effect also scaled with the individual's sensitivity to reward. This is consistent with previous observations that sensitivity to reward (as indexed by the SPSRQ) may reflect inter-individual variability in the structure (see Barros-Loscertales et al., 2006) and function (Adrian-Ventura 2019) of key hubs of the reward network (e.g., VTA, ventral striatum, orbitofrontal cortex), possibly modulating their influence on M1 activity during reinforcement skill learning. More research is required to better understand inter-individual factors shaping behavioral and neural responsiveness to reward during motor learning, an aspect that could be promising to determine which patients could benefit from reward-based motor rehabilitation protocols. Overall, our data suggest that the early reduction of neural variability in motor cortex may be an important mechanism underlying the benefits of reward on motor skill learning." We have now also added a summary statement in the Results section:

We state (page 23): "Overall, our findings indicate that reward coupled with performance feedback during training enhances rate of skill learning with a significant reduction in early-training MEP variability which correlates to individuals' sensitivity to reward scores." Discussion 6) Explain the clinical benefits of your work. For example, how could these findings be applied in rehabilitation settings? Discuss how rewards could be tailored to individual sensitivity levels to optimize recovery.

Authors' response: Thank you for this point. We have added this in our Discussion now.

We state (page 27): "Overall, our findings provide neurophysiological support for the incorporation of motivational cues in motor rehabilitation as recently attempted (Widmer et al., 2022, Therrien et al., 2016). More specifically, reward cues could be integrated in innovative technologies for motor restoration such as virtual reality, serious gaming or rehabilitation robots. In addition, these findings suggest that pre-screening patients for reward sensitivity may help to stratify patients according to their responsiveness to a reward-based rehabilitation protocol." 7) Address the limitations more thoroughly.

Authors' response: We have expanded the section on limitations in the Discussion.

We state (page 27): "Finally, we would like to address some factors that could have influenced our results, and the scope of these findings in the context of human motor skill behavior. First, our study could not be fully optimized to isolate subtle changes in UDP. Unlike previous studies on UDP, our force modulation task involved isometric, and not ballistic, movements and the training direction was not necessarily opposite to the TMS-induced movement direction as classically done in other work (Classen et al., 1998; Duque et al., 2008). Still, our study supports the view that even classical skill force modulation tasks can induce UDP (Mawase et al., 2017). Second, our study involved young healthy participants, and therefore future work is needed to assess if such reward-based effects on neurophysiology can be generalized to other populations such as older adults who typically exhibit reduced learning abilities (Maceira-Elvira et al., 2022). Third, while we utilized monetary reward in our study, effects of other forms of extrinsic rewards (e.g., social reward, Sugawara et al., 2012) needs to be explored further to obtain a better understanding of factors that drive human motor skills. Furthermore, this is not necessarily a limitation, but rather a deliberate choice to focus on resting-state measures, and as mentioned earlier, it would be fascinating to explore how CSE evolves dynamically during the task involving a motivational context with reinforcement feedback." ADDITIONAL COMMENT- We have made the title of our study concise, and would like to use the following on the title page- "Effect of extrinsic reward on motor plasticity during skill learning"
